# Managing disabled workers due to occupational accidents in Indonesia: a case study on return to work program

**DOI:** 10.1186/s12889-023-15930-2

**Published:** 2023-05-24

**Authors:** Arie Arizandi Kurnianto, Haitham Khatatbeh, Viktória Prémusz, Zsolt Nemeskéri, István Ágoston

**Affiliations:** 1 Doctoral School of Health Sciences, University of Pécs, Pécs, 7621 Hungary; 2 Department of Nusring, Faculty of Nursing, Jerash University, Jerash, 26150 Jordan; 3 Department of Cultural Theory and Applied Communication Sciences, Faculty of Cultural Studies, Teacher Training and Rural Development, University of Pécs, Pécs, 7633 Hungary

**Keywords:** Disabled workers, RTW Program, Occupational accidents, Qualitative research

## Abstract

**Background:**

Limited research and studies prove the usefulness of case management for persons with disabilities, which helps disabled employees recover their dignity through medical vocational, and psychological rehabilitation in underdeveloped countries’ Return To Work (RTW) programs.

**Methods:**

This qualitative case study design involved semi structured interviews with case managers as the primary data source, supplemented by secondary data from BPJS Ketenagakerjaan. Data analysis utilized QDA Miner Lite and Python with ArcGIS integration for descriptive visualization.

**Results:**

The RTW program of BPJS Ketenagakerjaan has already adopted ILO’s fundamental suggestion, which creates two central themes: internal aspects that are essential to the RTW framework and external variables that impact RTW practice. The key themes produce six main pieces to discuss further: personal skill, personal literacy, providers, guidelines, authorities, and stakeholder support.

**Conclusion:**

Return to Work Program benefits companies, and the implementation of a career development service or a partnership with non-governmental organizations guarantees that disabled employees who cannot return to work with their former employers are still in the global economy.

**Supplementary Information:**

The online version contains supplementary material available at 10.1186/s12889-023-15930-2.

## Introduction

Case management services for the disabled have experienced enormous development and implementation. The term “case management” refers to a series of procedures that are performed systematically and involve management, engagement, connectivity, and evaluation [1]. The rising incidence of industrial injuries has prompted case management to establish integrated health healthcare for at-risk employees. Within the context of case management, an effective theoretical framework for Return-to-Work (RTW) programs should be based on a biopsychosocial model based on ICF that considers the physical, psychological, and social needs of employees who have been injured or disabled, aiming to enhance worker capabilities and provide workplace accommodations to accommodate physical or psychological limitations. This approach goes beyond treating physical injuries or disabilities and also addresses the psychological and social aspects of the employee’s well-being, ensuring a holistic and comprehensive approach to RTW programs within the case management context [2, 3].

Injury and illness in the workplace likely had far-reaching cultural effects. The victim’s ability to earn a living may be compromised due to these accidents. The employees, in this case, are losing revenue due to their reduced capacity to do their duties or manage them. The only way for this is if an employee sustains an injury or develops an illness while working. It would have been even more devastating if the injured worker was also a primary income for the family. The employees may be permanently disadvantaged because of the nature of their disability. Each subsequent session of intensive therapy and rehabilitation is marked by a return to depression and a lack of interest in life because of the radical changes that the treatment has wreaked. Workers with disabilities can benefit much from rehabilitation and therapy, and they can also benefit significantly from the support and assistance of friends [4].

In addition, the Indonesian government has also recognized the significance of creating a program and strategy that might preclude people with disabilities (PwDs) from becoming forcibly removed from the workforce. The latest numbers indicate that the number of people with disabilities who are marginalized or removed from the job force exceeded 7 million in 2016, 3.74% of the estimated total of people with disabilities in Indonesia [5]. The amount would be significantly more significant when injured people who have already worked lose their jobs due to a work injury. There is no regulation or government entity to enable them to remain in the global economy. The Return-to-Work program is another approach to this challenge which allows workers who have suffered an injury to be ready to work and get the proper treatment and recovery.

The productive PwDs between 15 and 65 years old have been classified as active or inactive. The phenomenon of people with disabilities being excluded from the job force has exceeded a substantial portion compared to people with disabilities who are involved. Inactive PwDs refer to those who do not undertake household tasks or are in a time of studying yet are not even on the job force. Due to the limited opportunities for people with disabilities to work in formal sector occupations, the informal sector is the most significant preference for PwDs [6].

Besides that, despite the impairment, the disabled person typically gets a smaller wage than workers without disability [7]. Naturally, physical disability can be hereditary or perhaps due to an accident or disease [8]. Unlike a genetic or hereditary disability, people who suffer a disability due to an injury require special treatment to deal with their mental health. Therefore, workers who suffered injuries due to an occupational accident or disease should be included in the comprehensive rehabilitation engaged in the RTW program [9].

The RTW program aims to assist disabled workers and ensure that their situation does not disrupt them from the productive job force to the underemployed [10]. Heretofore, the RTW method concentrated only on vocationally medical rehabilitation. Rehabilitation is perceived to be the key factor in deciding the effectiveness of the RTW program. Nonetheless, other factors have brought in the beneficial impact of the RTW program, like ambient conditions and improvements in the workplace, which could be appropriate for employees with disabilities [11].

Indonesia had just initially begun the Return To Work Program, in which the development of the occupational accident benefits program (Jaminan Kecelakaan Kerja, JKK) has previously limited coverage. Initially, the Indonesian National Agency for Social Security on Employment (BPJS Ketenagakerjaan) managed the occupational accident benefit [12]. Notwithstanding, under the current regulation, solely formal workers can get admittance to the Return to Work Program. This includes companies that have made an agreement with BPJS Ketenagakerjaan to involve their employees in the program once the workers occasionally suffer from occupational accidents or disease and potentially have disabilities. Recently, the benefit of occupational accident insurance was cash-benefit and also medical treatment based on medical needs. Along the way of the RTW process, Case Managers of BPJS Ketenagakerjaan will assist the injured worker in starting from the emergence of the accident that defines the RTW’s plan continuously prior to injured workers getting fit to work [13].

Nevertheless, for completeness of the beneficial objectives of the RTW scheme, a concern has been established whether the RTW and new legislation could substantially allow disabled workers to continue in the same job as they had before the accident. The study addressed the advantages, deployment, and hurdles of the RTW mechanism. The importance discussing the importance of the new strategy and its implementation of the new strategy to develop the RTW program would include an explanation of the magnitude to which the RTW program assists disabled workers after suffering an occupational accident.

On that objective, a return-to-work program should have been implemented to reduce potential risks which might occur. In order to support employees who have been injured or disabled as a residue of injury and workplace disorder, a variety of specific aspects must be met by the social security company. For instance, the expense of medication, medical insurance, medical benefits the worst, or her wages, and the allowance for dependents if the breadwinner dies due to an injury at work or an accident. The return-to-work service is also important to ensure that the disabled worker still maintains his or her salary after retirement and will return to work for their former employer.

In comparison, the return-to-work initiative often offers more advantages for employees and employers as it encourages healing, decreases turnover, delivers rehabilitation, and, therefore, can help the employer sustain profitability against failure. Earlier research concentrates mostly on the usefulness of the return-to-work initiative and early involvement in the return-to-work process. Investigation revealed that the Return-to-Work System tends to minimize work detentions or days off for disabled workers. In the meantime, early action would help not just the employer but also the employees themselves.

Through early detection, the injured worker may be easily returned to work very rapidly as feasible. The interpretation of the Dutch RAT program seems pessimistic, which describes the management and employees confirmed that the RTW program provided even less assistance to the RTW participants [14]. Concurrently, empirical evidence that straightforward information and a scheme of return to work and occupational accident benefits offer the injured workers a wonderful understanding. The outcome of an empirical investigation with 141 disabled workers involved in the trial in which 72.3% of injured workers reported that being encouraged by the RTW program had an effect on their understanding of the return to work program [15]. In addition, the perception that colleagues and the company have of the disabled worker is also a crucial factor in the effectiveness of the return to work program. Upon reaching a certain threshold, RTW can be used as a proxy for the success of the healthcare and rehabilitation provided.

In order to address the potential challenges and benefits of RTW programs for disabled workers, this study aims to investigate the effectiveness of the current RTW program implementation in managing disabled workers due to occupational accidents in Indonesia, based on the perspectives of stakeholders. This will be achieved by conducting a comprehensive data collection process to gather information on various aspects of the RTW program, including its advantages, challenges, and impact on promoting the reintegration of disabled workers back into the workforce. The findings of this study will provide insights into the strengths and weaknesses of the current RTW program and contribute to the development of strategies for improving the management of disabled workers in Indonesia.

## Method

### Design

This case study was used in a qualitative research study. The RTW program is a conceptual framework for disability management [11, 16, 17], which in Indonesia involves occupational rehabilitation (medical, vocational, and psychosocial) programs. The case study is best for this study because it focuses on exploring the setting, conditions, and contexts [18] that happen along this existing program.

### Study context

This study was conducted through the Indonesian National Social Security Agency for Employment (BPJS Ketenagakerjaan) in a subset of the regional and branch offices, with the highest RTW cases standing as the statistical sample.

### Participants

The sampling approach consisted of strategic, criterion-based, and purposive random sampling procedures. This research has conducted a semi-structured interview by interviewing some case managers (CM) in some branches or regional offices of BPJS Ketenagakerjaan who actively engaged in certain cases of occupational accident that included in the RTW process to find in-depth insights into the implementation of RTW by BPJS Ketenagakerjaan and to identify a loophole in the RTW framework as more of an area for strengthening the RTW program throughout the years ahead. The interview will complement the study and provide wider knowledge and also perspective into the framework of the return to work program organized by BPJS Ketenagakerjaan. Eventually, indeed the findings of the initial interview that was gathered will be interpreted and grouped into particular subjects related to the research topic to define the significance of the data. In Table 1, we listed the characteristics of the respondents.

**Table 1:** Characteristics of respondents

Variables	Number of respondents (N = 11)
Age category	
30–35 years old	5
36–40 years old	4
40–45 years old	2
Gender	
Male	3
Female	8
Job Location	
Branch office	10
Regional Office	2
Level of Education	
Undergraduate	8
Postgraduate	3
Work Period	
3–5 years	1
6–9 years	3
10–14 years	6
more than 14 years	1

### Data collection

Interviews using a semi-structured format were carried out between February 2nd and February 26th, 2021. Prior to conducting the interviews, we sorted and filtered the offices by the top 10 highest claim rates and RTW based on data we got from BPJS Ketenagakerjaan about the distribution of claim rates for work-related injuries from 2014 to 2019. Case managers who were responsible for RTW at the chosen office will be informed about the study and invited to the interview if they are interested in participating. Due to the restrictions imposed during the COVID-19 outbreak, the interviews were carried out online. The.

### Data analysis

The outcome of the interview will be examined through data collection. Moreover, claim data from BPJS Ketenagakerjaan will be shown descriptively by integrating Python with ArcGIS. Based on grounded theory, our method of textual analysis for the semi-structured interview was exploratory and empirical. We looked for consistency in how these people went about the return to work. We also analyzed the data from the initial interviews conducted to decide how we proceed with the rest of the questions. Combining this strategy with the themes that were included in the theme’s rundown allowed us to successfully design a framework for the script.

## Results

### Overview of Indonesia’s adaptive return to work program

Implementing the Return to Work in Occupational Accident Benefits Program initiated by BPJS Ketenagakerjaan in 2015 began with a learning process in Malaysia and Germany. In the learning process, it was found that in its implementation, comprehensive monitoring and evaluation must be carried out periodically and continuously to ensure that the performance of the RTW program is very effective and beneficial for workers and employers.

In order to anticipate failure in the mentoring process to return to work, BPJS Ketenagakerjaan, through its case manager since 2017, has been assisting and monitoring RTW participants who have returned to work to determine the readiness of these participants in their fit to work, family support, work suitability with disability conditions, and environmental support. Work-related to accommodation that supports the limitations of the worker and the support of family and co-workers.

The Occupational Accident Benefit (Jaminan Kecelakaan Kerja (JKK)), which is organized by BPJS Ketenagakerjaan, covers healthcare services based on medical needs in cash compensation and educational scholarship in which will be granted for two dependent children of the insured person who dies or having a permanent disability as a result of an occupational accident. In addition, BPJS Ketenagakerjaan began to implement RTW in July 2015 with appointed eleven pilot project cities in Indonesia. These eleven regions were chosen because it is a densely populated industrial area, a labor-intensive tourism industry, and has many accident cases work that has the potential to participate in the Return to Work Program.

Deutsche Gesetzliche Unfallversicherung (DGUV), the occupational accident insurance agency in Germany in 2012, reported 2.6% of work accident cases could be managed by rehabilitation [19]. However, in Indonesia, since the implementation of RTW program until 2019, the number of companies committed to supporting RTW program has reached 68,824, and managed to handle 901 workers who have now returned to work to support their families.

**Fig. 1:**
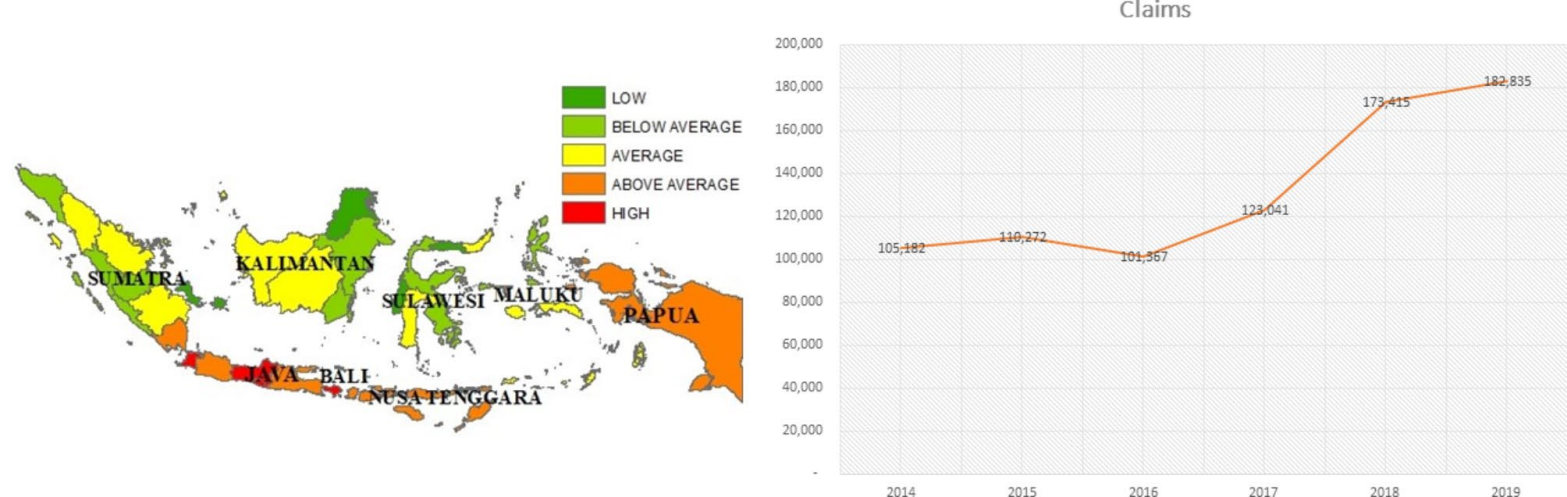
The distribution of the total number of claims for occupational accident insurance

**Fig. 2:**
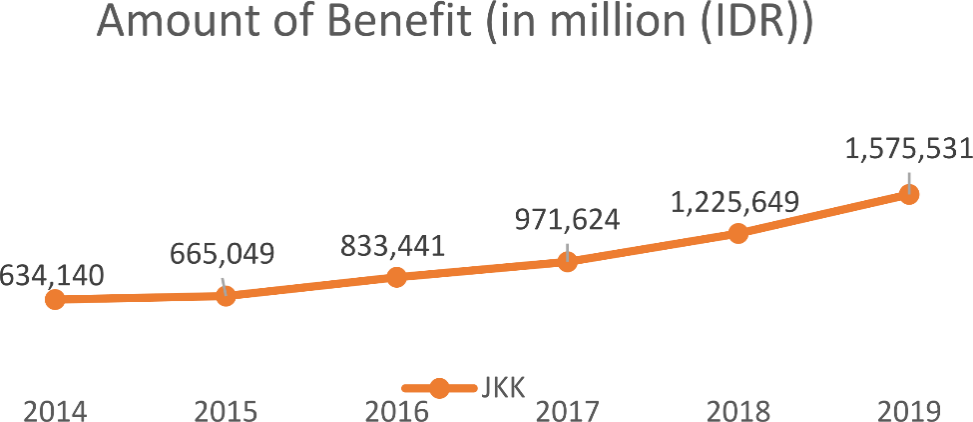
The total amount of benefit for occupational accidents

The line graphs in Figs. 1 and 2 illustrate the total number of claims and benefit for the employment injury protection program, which was described over six years from 2014 to 2019. The number of benefits is measured in currency Rupiah (IDR). Overall, the number of claims and compensation increased in six years of observation. The period of measurement was plotted to compare the time for benefit transformation before the implementation of Return To Work and afterward.

Total claims have been on the decline during the last six years, while the number of benefits received has been increasing. Moreover, an interesting variable that needs more exploration is the wide of protection covered in the mean time period. Interestingly, 2014 was the period in which a limited number of coverages was only IDR 20 million for one case without a return-to-work program. After 2015, the number of in-cash benefits climbed steadily to reach 1,575,531 million rupiahs in 2019. Meanwhile, there was a declining number of claims in 2016, at about less than 8%. However, the number of cases went up to reach a peak in 2020 of 182,835 claims. In the last five years, there are some developments of benefits have been implemented by BPJS Ketenagakerjaan, such as topping up the maximum allowable benefit from 20 million to unlimited based on medical needs and commencing the return-to-work program for workers suffering from occupational accidents or occupational disease.

### Participants of the RTW program and have returned to work

**Fig. 3:**
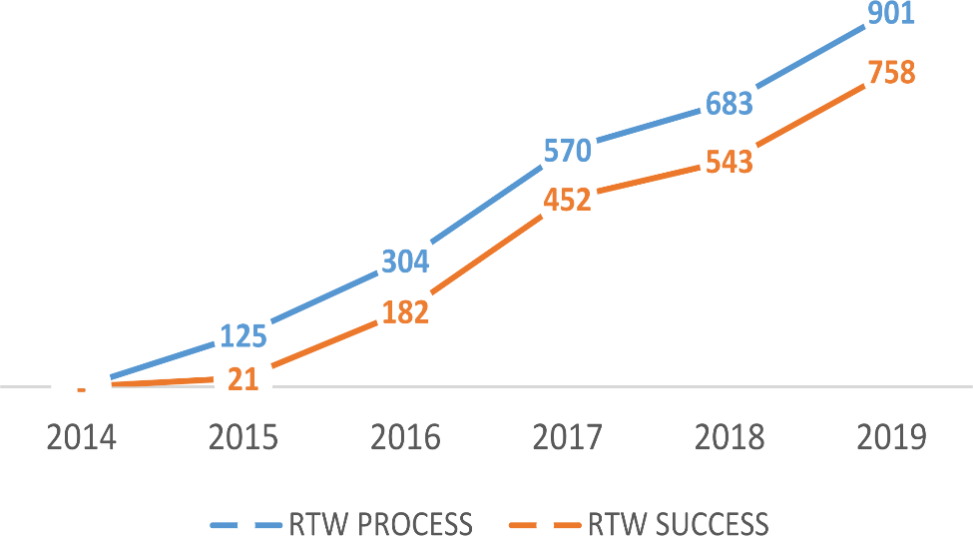
The distribution of workers who participated in the RTW program

According to the data presented in the line graphs in Fig. 3, there were only 125 participants in the RTW program in early 2015, with only 21 individuals returning to work, representing a 16.8% return rate. Over time, the implementation of RTW has become increasingly trusted, and as of December 2019, 901 participants have joined JKK RTW, and 758 participants have returned to work, or 85% have returned to work. In terms of participants who took part in the RTW program, it increased by 7.75%, and the increase of participants who had returned to work increased by 39.2%. In this case, 119 participants had not returned to work yet, in which were in the process of medical rehabilitation and job training, while 24 participants, or 16.43%, were categorized as having not succeeded in returning to work.

The semi-structured interviews have been analyzed, which resulted in the definition of two key themes and subthemes (Table 2). The specific remarks and excerpts provided by the interview participants would be provided in each of the key subtopics. Our study utilized a grounded theory approach, where the themes and sub-themes emerged from the data itself, based on the verbal answers provided by the case managers. As the study aimed to explore the perspectives of informants on the implementation of RTW program for disabled workers due to occupational accidents in Indonesia, the themes and sub-themes were generated from the data analysis process.

**Table 2:** Key Factors during the Implementation of the Return to Work Program

No.	Main theme	Subthemes	Quotation
1	Case Managers	Personal skills	“The solutions came in to argue sometimes, not only communication skill but our knowledge and experience are also coveted because approached them to emphasize their decency can be regained through the RTW program.”
		Personal literacy	“To assist a patient with disability to join RTW program is very challenging as we need comprehensive knowledge about case management to be able to engage integrative framework used to examine, schedule, enforce, organize, evaluate alternatives and solutions”
2	Barriers of implementation	Providers	“We often need to keep moving find a hospital or clinic to meet another specialist who has enough time and patience to explain the condition of the patient who is involved in the return to work scheme.”
		Authorities	“Another challenge is to constrain perceptions for employers to enable workers to be a part of the RTW program as we are only giving a recommendation. The authorities are on the employer’s hand.”
		Guidelines	“Regulations cannot explicitly be introduced to include workers in the RTW program, but employers’ and workers’ consideration and government support must be given for the provision of integrated, comprehensive rehabilitation services for workers with disabilities due to occupational accidents or disease.”
		Stakeholder’s support	“Even though there are regulations, not all companies are willing to participate their employees in the RTW program. They think this initiative will take a long time, so it’s best to hire new people who have the same skills.”

Essentially, the Return to Work Program in BPJS Ketenagakerjaan entails three phases framed in a case management cycle: identification for early tracing to a case of an occupational accident or occupational disease, evaluation, and the establishment of an adequate strategy with key stakeholders.

## Discussion

Systematic case management is an important aspect of any comprehensive RTW program, but recent study have found more evidentiary support for less intensive approaches [20]. Nonetheless, it is important to have a clear system in place for case management, including the identification of barriers to RTW and appropriate interventions. Effective case management is crucial for ensuring successful RTW outcomes. One aspect of a comprehensive RTW program is systematic case management, which plays an important role in ensuring successful RTW outcomes [21, 22].

According to several systematic reviews, complex RTW programs that include a workplace component and the use of an RTW coordinator may increase RTW rates and be particularly effective for individuals with musculoskeletal disorders [23]. Therefore, implementing a systematic case management approach with a well-trained RTW coordinator can lead to better outcomes for employees with occupational injuries or disease [24]. Moreover, research indicates that professionals working in specialized reintegration services are best suited to facilitate the job placement process of long-term sick-listed employees.

### Dynamic of case manager support in the RTW program

#### Personal skill

The number of assisting cases must be spread throughout Indonesia. Initially, there were only 14 case managers, but since 2019 there have been 81 case managers compared to 2015. They are in charge throughout branch offices of BPJS Ketenagakerjaan, in which depend on the risk of work accidents is quite high and causes disabilities in their areas of work. The case manager should have multidimensional skills to reliably determine the needs of the patient.

#### Personal literacy

Capabilities of case managers assisting PwDs during the Return to Work program presume as the internal factor which divided into interpersonal skills and literacy about case development that they handled [6] [25]. The key point to be convinced of is ‘assisting’ during the program, not only defined as accompanying the PwDs but also leading the plan to run the RTW program in order to achieve the expected outcomes in accordance with the initial plan.

### Barriers during the implementation of return to work in Indonesia

#### Providers

In addition, the external aspect that was substantially affecting the efficiency of the return to work program in Indonesia is the capacity of providers to return to work program. Not only in terms of infrastructure, services, and benefits managed by providers but also in terms of stakeholder literacy concern to the return to work program, the effectiveness and results to be obtained in RTW also have a substantial impact [26].

Not even all hospitals in Indonesia have an installation of medical rehabilitation facilities to carry out the RTW program. In fact, not all healthcare professionals have the assurance of the return to work program administered by BPJS Ketenagakerjaan. The outcomes purpose of integrated rehabilitation in RTW program is to strengthen the capacity of workers with disabilities to archive fit to work eventually [27].

#### Regulation

However, other more essential factors are authorities and guidelines, which are supported by government regulation. The company also has complete authority to enable the employees to join the Return to Work program. In this scheme, it is expected that the business enterprise would be able to reconsider the competitiveness aspect of workers with disabilities by placing them back in line with their work capability. One of the requirements that have to be met is that employers and workers are willing to submit a declaration of consent to engage in the return to work program [28].

#### Authorities

The managerial declaration made by a company is intended to guarantee that workers are assured that they will be warmly welcomed to work in the company where they work after just actively engaging in the program. Moreover, while it is expected that employees who experience an injury at work should be able to work again, the existing rules on the operation of the Return to Work Policy do not explicitly govern whether or not the workers involved may work in the same workplace. Workers can work again and do the same job, and they can also do some work that is adapted to the circumstances of an injury at work.

#### Stakeholders’ support

In 2015 there were 2,080 companies that supported the RTW program, which continued to increase, and up to September 2020, there were 70,820 employer supports or an increase of 3,404% compared to 2015. This support is a manifestation of the employer’s commitment to supporting the RTW and willingness to re-employ disabled workers from both their own companies and other companies. This support is still being improved because recently there are only 10.51% of the total employers participated in BPJS Ketenagakerjaan. The challenges in convincing companies to ensure that workers with disabilities can return to work are still enormous.

According to recent regulations, this is not mandatory for employees experiencing occupational injuries and occupational diseases experiencing disability to be eligible for a return to work program [28]. Meanwhile, Article 153(1) of Law No 13 of 2003 forbids companies to abandon work on the basis that employees are seriously injured, disabled due to injuries at work or sickness due to work affiliations and, according to the doctor’s statement, have not yet been able to retrieve. In this regard, as Indonesia’s national agency for social security on employment, which is a government representative, BPJS Ketenagakerjaan should be supported by a regulation under the Ministry of Manpower in Indonesia which regulates the role of the Government and BPJS Ketenagakerjaan to provide support for workers with disabilities and who are unacceptable to the company. This is to support law enforcement related to Article 153, paragraph 1 (j) of the Manpower Law and similar provisions in the Law on Persons with Disabilities [29–31].

The aspect of professional social security provided by the government to ensure that civilians fulfill the fundamental needs of a decent quality of life is a form of social security. Moreover, social welfare that concerns social protection, or protection against known social conditions, including poverty, old age, disability, unemployment, family and children, and others, recently have been deliberated as the essential concern in the UN Declaration 1948 on Human Rights and the ILO Convention No.102 1952 [32].

In general, occupational accident benefits programs are developed in the form of employment injury benefits or health insurance for the workforce. However, the reality remains that the health service process cannot be separated from health financing. Health costs are the number of funds that must be provided to organize and or take advantage of various health efforts required by individuals, families, groups, and communities. Solid, reliable, and secure healthcare contexts equalize a crucial role in providing health services in order to achieve numerous essential health policy goals in the world, including equal access to healthcare and regulatory compliance [33, 34]. Accordingly, healthcare changes in a state should also impose an important emphasis on national healthcare policies to ensure the appropriateness, fairness, quality, and efficacy of the healthcare expenditure system.

Moreover, occupational accident benefits programs in Indonesia have been formulated to be in-cash benefits and in-kind benefits. It gives protection against accident risks taking place under employment relationships, including an accident on the way to the workplace from the house. The protection starts from the way to the workplace, leaving the workplace to return home, working at the workplace, and during taking duty travels. Furthermore, the in-cash benefit provided includes the amount of money as compensation for the occupational risk, which will be counted by the reported wages. Meanwhile, the in-kind benefit engages with the protection during the curative and rehabilitative services in health care providers. The cost will be unlimited medical treatment as medically necessary [35].

Occupational accident benefits complemented by RTW program, in which defined as the resettlement of workers who are sick and have not been able to work either in the short or long term so that they can return to work immediately. In the guidelines for the International Social Security Association (ISSA) [36], which is a reference for countries that already have a social security system and have implemented the RTW program, it is stated in guideline 9 that the RTW program is based on a bio-psycho-social approach that combines medical, psychological aspects and social as described in the framework of The WHO International Classification of Functioning, Disability, and Health (ICF) [37]. The rate of the Return To Work program engaged with a concept of returning early to work safely and being able to maintain or stay with the job [38]. Return to Work is a term when a person enters the back to work phase, but it does not guarantee success because many workers return to work after experiencing disabilities due to work accidents but cannot survive their jobs [11]. The program mainly involves medical rehabilitation, vocational rehabilitation, and psychosocial rehabilitation.

### Challenges during the outbreak of COVID-19

In Indonesia, to bring specialist health services closer and improve the quality of health services in healthcare facilities, especially during the outbreak of the COVID-19 pandemic, various efforts are being made, one of them using information technology in the health sector in the form of consultation services between health care facilities through Telemedicine. The Government of Indonesia has established a new regulation, namely the Regulation of the Minister of Health of the Republic of Indonesia Number 20 of 2019, concerning the Implementation of Telemedicine Services Between Health Care Facilities [39]. The Return-to-Work Program managed by BPJS Ketenagakerjaan in its service has implemented Telemedicine as one of the comprehensive services of vocational-based telerehabilitation, often combined with behavioral therapy, during the COVID-19 pandemic. The use of Telemedicine has been recognized by the college of the relevant medical profession to initiate or treat people with disabilities through Telemedicine without the need for direct examination.

Numerous programs have been attempted in Indonesia in regard to the existence of Return To Work, including the digitalization of RTW support networks, especially for stakeholders in the Centre of Services for Occupational Accidents and Return-to-Work. Telemedicine can be used to improve the efficiency and efficacy of trauma treatment delivery. In addition, Telemedicine is used in the treatment of trauma patients. The advantages of Telemedicine in RTW are strongly intertwined with substantial support from government issues regulation related to Telemedicine, such as the Regulation of the Minister of Health and the Decree of the Minister of Manpower. Moreover, the diverse archipelagic state encourages BPJS Ketenagakerjaan to assign a trustworthy case manager to handle RTW cases with Telemedicine in branch offices. Furthermore, in comparison to traditional venues, Telemedicine has enhanced the availability of competent, time-efficient, and cost-effective multidisciplinary medical expertise sessions that benefit patients regardless of location.

However, regulatory challenges are related to the difficulty and cost of obtaining licensure across multiple regions and privileges at multiple facilities. In regards to the issue, major financial barriers may be raised as Telemedicine need a reliable broadband connection and broadband mobile communication technology. Another downside that may be considered is the cultural barriers occurring from the lack of desire, or unwillingness, of some patients or healthcare workers to adapt clinical paradigms for telemedicine applications.

In terms of education, BPJS Ketenagakerjaan also conducts regular intensive programs for stakeholders, BPJS Ketenagakerjaan case managers, and RTW support company partners in the form of webinars related to disability management. The RTW milestone development plan is to initiate inclusive job service, in which we will provide the connection as the Hub Supply and Demand for Disables workers and companies as a measure of success in the RTW framework.

### Implication and limitation of the study

#### Implication for future research

Future research could examine the impact of increasing the number of case managers in the Return to Work (RTW) programme in Indonesia. Researchers could investigate how the increased number of case managers has affected the quality and efficacy of the RTW programme, as well as the outcomes attained by workers with disabilities. In addition, future research could focus on the specific multidimensional skills case managers need to effectively determine the requirements of patients during the RTW programme, as well as how these skills can be further developed or improved to increase the program’s overall success.

#### Limitation of the study

One of the limitations of the study is that the sample only includes BPJS Ketenagakerjaan beneficiaries in Indonesia, which represents one country perspective. Therefore, the results may not be generalizable to other regions or countries, including case management for disabled workers worldwide. In addition, the results of this research have not been explained from the health perspective of case managers or participants in the RTW program who are workers who are disabled as a result of work accidents. Thus, it is important to investigate in further studies to observe the quality of life and work ability index of participants after participating in RTW program. Furthermore, an economic assessment of the RTW programme in Indonesia might be studied in the future to determine the full impact.

## Conclusion

The Return to Work (RTW) Programme plays a critical role in effectively managing employees with disabilities resulting from occupational injuries in Indonesia. The success of the Indonesian RTW program hinges on the personal skills and literacy of case managers, as well as addressing implementation barriers such as provider availability, employer and government support, and stakeholder perception. The provision of comprehensive rehabilitation services for workers with impairments due to industrial accidents or illnesses is imperative, and regulatory measures alone are insufficient to ensure active participation of workers in the program. Therefore, further research will be required to investigate the outcomes of the RTW program for disabled workers, analyze the experiences of case managers, and evaluate the economic effect of the program.

## Electronic supplementary material

Below is the link to the electronic supplementary material.

Supplementary Material 1

## Data Availability

The authors state that all data validated by the report’s findings are included in the document.
