# Supplementary material for: Managing disabled workers due to occupational accidents in Indonesia: a case study on return to work program
Source: BMC Public Health. 2023 May 24;23:943. doi: 10.1186/s12889-023-15930-2 (PMC10206580; doi:10.1186/s12889-023-15930-2)
Supplement: Supplementary file 1 — Supplementary Material 1 [file 12889_2023_15930_MOESM1_ESM.docx]

Appendix. Questions that were used as a guide throughout the semi-structured interview

| No | Theme | Question |
| --- | --- | --- |
| 1 | Profile | Inquiring about the respondent's profile, which includes name, gender, and job unit |
| 2 | General impression | What is your perspective as a Case Manager BPJS Ketenagakerjaan about Return-to-Work Program? |
| 3 | Challenge | According to your experiences and knowledge as a Case Manager at BPJS Ketenagakerjaan, what difficulties or challenges are regularly presented in your work region and nationally, especially those associated to the Return to Work program? |
| 4 | Engagement | How does BPJS Ketenagakerjaan's Return To Work program relate to the government/companies/workers/partners in your area? Please describe your latest encounter: Issues addressed, expected outcomes, or conversations' realization? |
| 5 | Utilization | How can the government/companies/workers/partners of BPJS Ketenagakerjaan (provider) improve the advantages of the Return to Work program? |
| 6 | Evaluation | How do you assess the attempt of Return to Work program to enhance services to the members of BPJS Ketenagakerjaan (good/bad, why)? |
| 7 | Improvement | How can BPJS Ketenagakerjaan enhance and optimize the Return To Work program? |
| 8 | Augmentation | What can/shall be done to encourage Return to Work participation? Probe : challenges, possibilities, stakeholders/parties to cooperate. |
